# Effect of stereotactic body radiotherapy on regional metabolic liver function investigated in patients by dynamic [^18^F]FDGal PET/CT

**DOI:** 10.1186/s13014-021-01909-z

**Published:** 2021-10-01

**Authors:** Michael Sørensen, Mette Marie Fode, Jørgen Baltzer Petersen, Marianne Ingerslev Holt, Morten Høyer

**Affiliations:** 1 Departement of Nuclear Medicine & PET, Aarhus University Hospital, Aarhus N, Denmark; 2 Departement of Hepatology & Gastroenterology, Aarhus University Hospital, Palle Juul-Jensens Boulevard 99, C116, 8200 Aarhus N, Denmark; 3 Departement of Oncology, Aarhus University Hospital, Aarhus N, Denmark; 4 Departement of Medical Physics, Aarhus University Hospital, Aarhus N, Denmark; 5 Danish Centre for Particle Therapy, Aarhus University Hospital, Aarhus N, Denmark; 6 Department of Internal Medicine, Viborg Regional Hospital, Viborg, Denmark; 7 Department of Genetics, Vejle Hospital, Vejle, Denmark

**Keywords:** Galactokinase, Metabolic liver function, Functional imaging, Liver cancer, Oncology

## Abstract

**Purpose:**

Stereotactic body radiotherapy (SBRT) is increasingly used for treatment of liver tumors but the effect on metabolic liver function in surrounding tissue is largely unknown. Using 2-deoxy-2-[^18^F]fluoro-d-galactose ([^18^F]FDGal) positron emission tomography (PET)/computed tomography (CT), we aimed to determine a dose–response relationship between radiation dose and metabolic liver function as well as recovery.

Procedures.

One male subject with intrahepatic cholangiocarcinoma and five subjects (1 female, 4 male) with liver metastases from colorectal cancer (mCRC) underwent [^18^F]FDGal PET/CT before SBRT and after 1 and 3 months. The dose response was calculated using the data after 1 month and the relative recovery was evaluated after 3 months. All patients had normal liver function at time of inclusion.

**Results:**

A linear dose–response relationship for the individual liver voxel dose was seen until approximately 30 Gy. By fitting a polynomial curve to data, a mean TD_50_ of 18 Gy was determined with a 95% CI from 12 to 26 Gy. After 3 months, a substantial recovery was observed except in tissue receiving more than 25 Gy.

**Conclusions:**

[^18^F]FDGal PET/CT makes it possible to determine a dose–response relationship between radiation dose and metabolic liver function, here with a TD_50_ of 18 Gy (95% CI 12–26 Gy). Moreover, the method makes it possible to estimate metabolic recovery in liver tissue.

## Introduction

Stereotactic body radiotherapy (SBRT) is increasingly used as a local ablative therapy of liver tumors with emerging evidence that it is effective and safe in both primary [1] and secondary liver cancer [2, 3]. Radiotherapy is, however, still underused for liver tumors and radiotherapy treatment planning is conservative with regards to doses and volumes to the liver, mainly because of sparse knowledge about the radiation tolerance of the liver. Based on surgical experience, it is recommended that at least 700 mL liver tissue be spared and receive a maximum total dose of 15 Gy when SBRT is delivered in 3–6 fractions [4]. It is also recommended that the mean radiation dose delivered to the liver do not exceed 15 Gy in patients without parenchymal liver disease and 13 Gy in patients with viral hepatitis and/or cirrhosis [4]. However, a precise dose–response relationship for liver tissue has not been established and the potential of SBRT in treatment of liver tumors is thus not fully utilized.

Galactose is metabolized in the liver by the cytosolic enzyme galactokinase and the galactose elimination capacity test provides a robust measure of metabolic liver function as demonstrated by its high prognostic sensitivity [5–10]. The fluorine-18 labeled galactose analogue 2-deoxy-2-[^18^F]fluoro-d-galactose ([^18^F]FDGal) is also metabolized by galactokinase and is used for functional positron emission tomography (PET) studies of regional metabolic liver function [11–14]. From dynamic PET scans of the liver with intravenous bolus injection of [^18^F]FDGal, so-called parametric images of the liver function can be created which provides voxel-by-voxel values of the hepatic systemic clearance of [^18^F]FDGal (*K*_met_, mL blood/min/mL liver tissue). *K*_met_ is a flow-independent measure of galactokinase capacity that provides an indirect measure of the maximum removal capacity of [^18^F]FDGal regionally in the liver [12–14] with low individual day-to-day variation [15, 16].

The aim of the present study was to quantify the effect of radiotherapy on metabolic liver function measured by dynamic ^18^F-FDGal PET/CT and to determine a dose–response relationship between regionally delivered radiation dose and metabolic liver function in patients treated with SBRT of liver tumors. Metabolic recovery was determined after 3 months.

## Materials and methods

### Patient cohort

One male patient with intrahepatic cholangiocarcinoma (IHC) and five patients (1 female, 4 male) with liver metastases from colorectal cancer (mCRC) were included in the present prospective study (Table 1). All patients had normal liver function, and none had parenchymal liver disease including cirrhosis. Inclusion criteria were patients deemed unsuitable for surgery or radiofrequency ablation, but eligible for SBRT, age > 18 years, and performance status < 2. Exclusion criteria were impaired kidney function, and pregnancy.

**Table 1:** Patient characteristics and treatment plan

ID	Sex/age	Diagnosis	Number of tumors treated	Previous local treatment	Σ CTV (cc)	Liver-CTV (cc)	Total dose (Gy)/fractions
1	M/59	IHC	1	–	67.7	2829.0	54/6
2	M/62	mCRC	1	–	16.1	1775.8	56.25/3
3	M/68	mCRC	1	RFA	6.3	3219.9	56.25/3
4	F/91	mCRC	1	–	44.1	1121.9	45/3
5	M/64	mCRC	3	RFA	25.0	2113.7	45/3
6	M/62	mCRC	2	Resection	1.2	1433.3	56.25/3

*M* male, *F* female, *IHC* intrahepatic cholangiocarcinoma, *mCRC* metastatic colorectal cancer, *RFA* radiofrequency ablation, *Σ* sum, *CTV* clinical target volume, *cc* cubic centimeters, *Gy* gray

### Stereotactic body radiotherapy (SBRT)

For SBRT, patients were immobilized in a stereotactic body frame (SBF: Elekta, Stockholm, Sweden) and a moderate diaphragmatic compression was applied for minimization of the respiration related movement of the liver. A CT-scan (Brilliance Big Bore, Philips Medical Systems, Bothell, WA) with intravenous infusion of contrast media (Visipaque 275 mg I/mL; 2 mL/kg body weight) with a delay of 70 s was performed for delineation of the clinical target volume (CTV). 3D conformal treatment plans were constructed with 5–8 static coplanare and/or non-coplanare beams with 6 MV formed by millennium multi leaf collimators (MLC) using the Eclipse treatment planning system (Varian, Palo Alto, CA). The CTV was defined as the gross tumor volume (GTV). Since all patients had little or moderate respiration related motion of the liver, a standard margin of 5 mm in the transverse plane and 10 mm in the cranio-caudal plane was added to create the planning target volume (PTV). For patients with mCRC, the dose prescribed to the CTV was 45 or 56.25 Gy in three fractions. In the patient with IHC, the dose was 54 Gy in six fractions due to the CTV’s proximity of the duodenum. The dose was prescribed as the mean dose to the CTV and the CTV was enclosed by 95% and the PTV by 67% isodose surface. All patients were treated on a Varian Clinac Accelerator equipped with on-board kV-imaging system (Varian Medical Systems, Palo Alto, CA). A cone-beam CT-scan was performed prior to each fraction and the vertebral spine on the CBCT was co-registered liver-to-liver on the treatment planning CT-scan. The diaphragm served as the primary fix point during the co-registration. Any positional error was corrected before start of treatment.

### [^18^F]FDGal PET/CT

Functional liver [^18^F]FDGal PET/CT was performed before treatment and 1 and 3 months after last treatment fraction. Before each scan, a small catheter (Artflon, Ohmeda, Swindon, UK) was placed in a radial artery for blood sampling. Next, the patient was placed in supine position in the PET/CT camera (Siemens 64 Biograph TruePoint; Siemens AG, Erlangen, Germany) and the liver was positioned in the 21.6 cm transaxial field-of-view. A low-dose CT scan (50 effective mAs with CAREDose4D, 120 kV, pitch 0.8, slice thickness 5 mm) was performed for attenuation correction of PET emission data. A bolus of 100 MBq [^18^F]FDGal dissolved in 10 mL saline was administered intravenously during the initial 20 s of a 20-min dynamic PET scan. [^18^F]FDGal was produced at our own radiochemistry laboratory with a radiochemical purity ≥ 97% [17]. PET data were recorded in list mode and reconstructed without resolution modeling (336 matrix, voxel size 2 × 2 × 2 mm^3^, 6 iterations, 21 subsets, 2 mm Gaussian filter, separate prompts/randoms, no time-of-flight) and corrected for radioactivity decay back to start of the PET scan. Arterial blood samples (frequent in the beginning, fewer later in the scan) were collected during the scan for measurements of arterial blood concentrations of [^18^F]FDGal (Packard well counter), which were corrected for radioactivity decay back to the start of the PET scan.

### Analysis of ^18^F-FDGal PET/CT data

Parametric images of *K*_met_ (mL blood/min/mL liver tissue) were created using the PMOD software (PMOD Technologies Ltd, Zürich, Switzerland). In short, the kinetic model fitted to data assumes irreversible trapping of ^18^F-FDGal in hepatocytes by phosphorylation in hepatocytes by galactokinase with quasi-steady state from 6 to 20 min after the bolus injection of [^18^F]FDGal [11–14]. Using the MIM Software Version 6.5 (MIM Software Inc, Cleveland, OH, USA), the parametric images were deformably co-registered liver-to-liver with the planning CT scan with same voxel size as the original PET images, and *K*_met_ values in regions receiving 0–5 Gy, 5–10 Gy etc. up to maximum dose (Table 1) were extracted. For the patient receiving six fractions, the doses were converted to biologically effective dose for three fractions using α/β = 3 for liver tissue using the equation for equivalent dose [18]:

$$\frac{{D_{1} }}{{D_{2} }} = \frac{{D_{1} /n_{1} + \alpha /\beta }}{{D_{2} /n_{2} + \alpha /\beta }}$$

where *D*_*i*_ and *n*_*i*_ designate total dose and number of fractions, respectively. For two different fractionation schemes, we thus find:

$$D_{2} = \frac{{ - \alpha /\beta + \sqrt {\left( {\alpha /\beta } \right)^{2} + \frac{{4D_{1} \left( {d_{1} + \alpha /\beta } \right)}}{{n_{2} }}} }}{{2/n_{2} }}$$

The solution yielding a negative dose was omitted. Individual dosimetric data are shown in Table 2.

**Table 2:** Individual dosimetric data

ID	V50 (cc)	V40 (cc)	V30 (cc)	V20 (cc)	V10 (cc)	D_mean_ (Gy)
1	60.7	114.8	195.2	365.0	644.5	8.2
2	33.2	68.1	170.4	328.6	472.3	8.8
3	15.8	28.4	50.3	144.1	452.9	4.3
4	–	51.4	109.1	170.6	266.6	7.2
5	–	79.3	152.5	241.6	380.9	6.5
6	5.2	9.1	14.7	22.9	46.9	1.4

All volumes are liver volume without CTV

*cc* cubic centimetres

The parametric images after 1 and 3 months were compared to the baseline scan and the relative *K*_met_ for each dose-interval was calculated.

A dose–response relationship between radiation dose and *K*_met_ was calculated using the 1-month [^18^F]FDGal PET/CT. *K*_met_ values for each dose-interval (*K*′_met_) were normalised to percentage using the equation [19]:

$$\left( {K_{{{\text{met}}}}^{\prime } - K_{{{\text{met\_min}}}}^{\prime } } \right)/\left( {K_{{{\text{met\_max}}}}^{\prime } {-}K_{{{\text{met\_min}}}}^{\prime } } \right)$$

where *K*′_met_min_ represents the individual minimum *K*_met_ and *K*′_met_max_ the maximal individual mean *K*_met_. This normalization assumes that liver tissue receiving a radiation dose of 0–5 Gy is normally functioning (100%) and liver tissue receiving maximum dose is non-functioning (0%). The latter assumption was supported by the fact that liver tissue receiving high radiation doses had *K*_met_ values in the same order as those in intra-hepatic areas previously treated with radiofrequency ablation, i.e. areas without [^18^F]FDGal metabolism. Data were normal distributed (Q-Q plot) and a polynomial equation (y = ax^2^ + bx + c) was fitted to the mean values. The 95% confidence intervals (95% CI) for the mean were calculated as mean ± 1.96 SEM, where SEM is the standard error of the mean. The polynomial model was chosen because of the shape of the dose–response curve (see Results). A mean TD_50_, defined as the dose at which the metabolic function was 50% of the maximum dose, was calculated with 95% confidence intervals (CI).

## Results

**Fig. 1:**
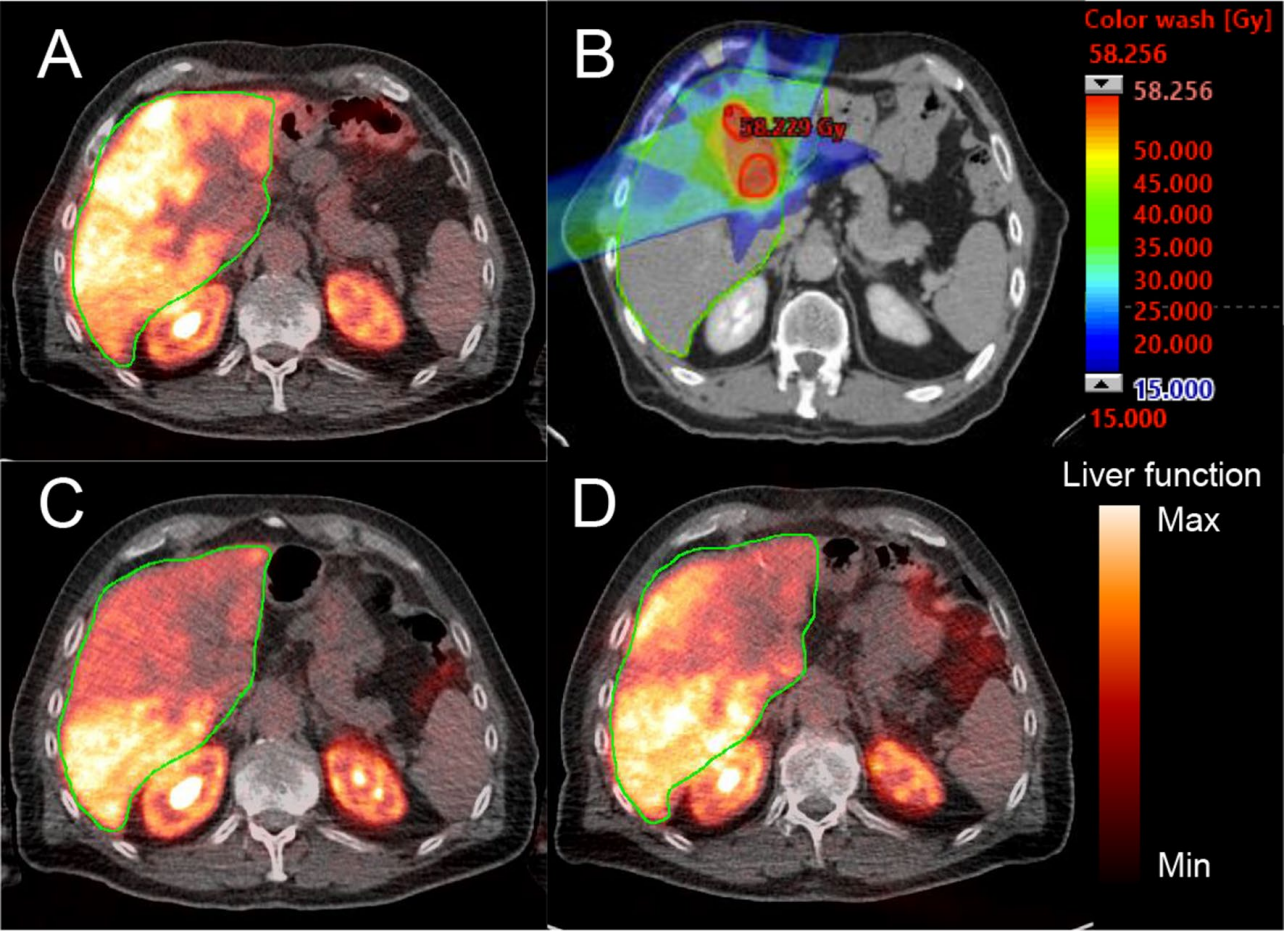
Example of parametric image of *K*_met_ (hepatic systemic clearance of [^18^F]FDGal, mL blood/min/mL blood). A is before SBRT of a colorectal liver metastasis; note that the tumour does not accumulate [^18^F]FDGal. B is the treatment planning CT with dose color wash of 15 Gy. C and D show parametric images after 1 month and 3 months, respectively. The liver is encircled by a green line and the color scale for the PET images show *K*_met_ ranging from 0.0 to 0.3 mL blood/min/mL liver tissue

Average time from baseline [^18^F]FDGal PET/CT scan to treatment start was 7 (range 5–10) days. Average time from first treatment to the 1-month scan was 40 (range 36–48) days and average time from first treatment to the 3-month scan was 97 (range 86–109) days. Visually, there was a clear decline in *K*_met_ of [^18^F]FDGal on images after 1 month, as shown in the example in Fig. 1. Three months after SBRT, the changes were not as marked (Fig. 1D) in accordance with the high regeneration capacity of the liver. Figure 1D illustrates the radiation fields and the dose color wash to the CTV and the normal liver tissue.

**Fig. 2:**
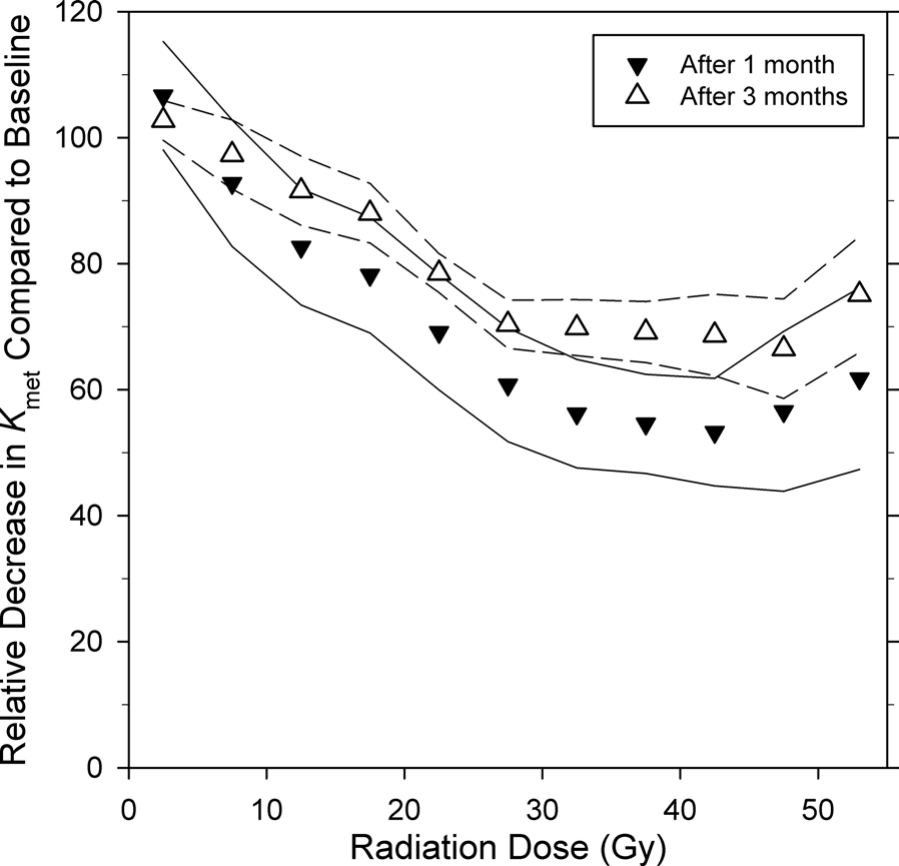
Mean *K*_met_ values relative to baseline values after 1 month (▾) and 3 months (∆) versus radiation dose (Gy) represented as mean for all six patients. The lines show the standard error of the mean (SEM) for 1 month (solid lines) and 3 months (dashed line)

Figure 2 shows the relative *K*_met_ values for each dose-interval after 1 month and 3 months. In three patients, *K*_met_ values in regions that received up to 20 Gy were higher than baseline (105–120% of baseline) after 1 month reflecting a metabolic reserve capacity of the liver. In areas receiving doses higher than approximately 20 Gy, *K*_met_ decreased to 30–40% of baseline values with increasing doses. After three months, *K*_met_ had normalized in low-dose regions (up to 10 Gy) and increased to approximately 50–85% of baseline values in regions receiving higher doses.

**Fig. 3:**
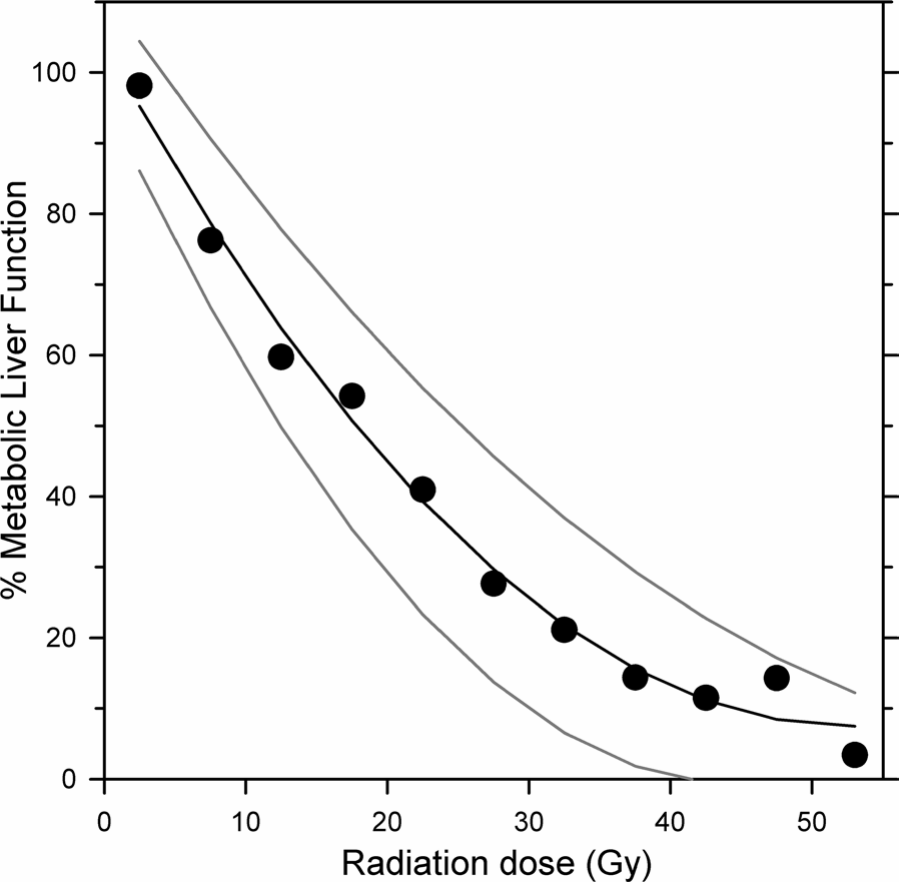
Dose–response relationship between liver function (percent *K*_met_, mL blood/min/mL liver tissue) versus radiation dose (Gy) with means (●), fitted mean (black line), and upper and lower 95% confidence intervals (grey lines) (B) using data after 1 month

Figure 3 shows the mean dose–response relationship between delivered radiation dose and decrease in metabolic liver function after 1 month. As seen, the relationship was linear until approximately 30 Gy and then became more horizontal. By fitting a polynomial curve to data, a mean TD_50_ of 17.8 Gy was determined with a 95% CI from 12.0 to 25.8 Gy.

## Discussion

Knowledge of the pathophysiological effects of radiotherapy on normal liver tissue surrounding the target tumor is essential for optimizing SBRT of liver tumors. The liver is known to have a large volume-effect as well as a certain metabolic reserve capacity, but the effect of radiotherapy on metabolic function is largely unknown. In the present study, we aimed to determine a dose–response relationship between radiation dose delivered to normal liver tissue by SBRT and metabolic liver function in terms of hepatic metabolic clearance of [^18^F]FDGal and found a mean TD_50_ of 17.8 Gy. This could have clinical potential for the individual SBRT planning.

Our data do not allow us to make a dose-complication rate relationship e.g. for development of radiation-induced liver disease (RILD) which would require a much larger patient cohort. Moreover, whereas RILD is primarily characterized by hepatic venous clotting [20], [^18^F]FDGal PET/CT measures the capacity of the galactokinase enzyme in hepatocytes and the dose–response relationship presented here accordingly reflects a direct effect on hepatocyte metabolic function.

Herfarth et al. studied local changes in the hepatic blood perfusion by multi-phase CT-scans in 36 patients treated by single dose SBRT. Based on the radiological patterns reflecting changes in contrast kinetics after contrast enhancement, focal radiation reactions occurred at a median of 1.8 months after SBRT with a median threshold dose of 13.7 Gy (range 8.9–19.2 Gy) [21]. This is in accordance with our results although it should be kept in mind that the Herfarth data reflects the vascular damage and not the metabolic changes. In the present study, the *K*_met_-values do not depend on hepatic blood flow but solely on galactokinase activity [12, 13]. It seems likely, however, that volumes with decreased perfusion also have decreased metabolic function. This is also supported by a dose-dependent decrease in portal venous blood flow after SBRT [22].

There are some limitations to our study. First, the metabolic reserve capacity of the liver is well described, and it has been shown that the galactokinase activity may increase per volume liver tissue after surgery in both rats [23, 24] and humans [25, 26]. In accordance with this, *K*_met_ increased in some of the subjects in the present study 1 month after treatment compared to baseline in areas receiving a low radiation dose. This is a potential disadvantage of the [^18^F]FDGal PET method, but it remains one of the only methods for reliable quantification of regional metabolic liver function using a validated method. Moreover, the potential effect on the dose–response calculation was corrected for by normalizing the individual data as described in Materials and Methods. It should also be noted that the baseline values are not subject to reserve capacity and the potential use of [^18^F]FDGal PET/CT in functional treatment planning (FTP, see below) is thus not affected by this. Second, the number of patients was relatively low, but the pattern of changes and the dose–response changes were very consistent, and we therefore found the number sufficient for a robust conclusion. In addition, the results are in line with our results in a phase I study using the [^18^F]FDGal PET/CT method to optimize the SBRT treatment plan of liver metastases [27]. Third, we only included patients with tumors in otherwise healthy liver tissue. i.e. non-cirrhotic livers and did not include patients with cirrhosis of the liver though the frequency of SBRT used in e.g. patients with hepatocellular carcinoma is increasing due to technological advances [28, 29]. Cirrhotic liver tissue is more vulnerable to radiotherapy than healthy liver tissue and the tolerance dose and recovery rate will most likely be lower than what we found in the present study [4].

Furthermore, we have previously shown that the liver function is more heterogeneously distributed in the cirrhotic liver than in the healthy liver [13] which underlines that careful treatment-planning is important in that subgroup of patients. However, a precise relationship between radiation dose and liver function in hepatitis virus carriers and patients with cirrhosis livers should be determined separately. Individual tolerance to radiation treatment in those patients is likely to also depend on other factors such as individual liver impairment, performance status etc. It should be mentioned that the present method using [^18^F]FDGal PET/CT for measuring regional metabolic liver function has been validated in both healthy subjects and patients with cirrhosis, and it is thus readily applicable for patients with parenchymal liver disease as well [12–14].

A fourth limitation of the study is the risk of mismatch in the deformable co-registration of the PET/CT scan at baseline, 1 and 3 months after SBRT and the planning CT. Motion management and the co-registration of images are factors that should be optimized in the future. Introducing fiducial markers as a surrogate marker of tumor has reduced the uncertainty but were not used in the present study.

An important future perspective would be to utilize [^18^F]FDGal PET/CT in individualized treatment of patients with primary and secondary cancer of the liver. Adaptation based on scans acquired during SBRT will not be meaningful due to the late tissue response and the short treatment course of SBRT. However, it seems likely that individualized dose prescription or FTP is possible with sparing of the best functioning sub-volumes of the liver on a pretreatment [^18^F]FDGal PET/CT during optimization of the treatment planning [27, 30, 31]. Moreover, techniques for liver-sparing are also warranted due to increased radio-sensitivity in some patients treated with the combination of SBRT and systemic therapy [32]. Accordingly, the indications of SBRT for liver tumors could be expanded to patients with reduced liver function and/or a larger tumor burden than possible to treat with SBRT at the present time point by introducing FTP. Including functional imaging such as the present method also allows for more precise predictions of tissue tolerance instead of modeling [33]. A prospective randomized, case-controlled study for evaluation of the effect of including functional imaging in treatment planning on post-SBRT morbidity and survival would be interesting but should preferably be multi-center for enough patients to be enrolled.

It should be noted that the radiochemical production of [^18^F]FDGal is similar to that of the common PET tracer 2-deoxy-2-[^18^F]fluoro-D-glucose ([^18^F]FDG) [17] which allows for a broad application of [^18^F]FDGal PET/CT. If onsite production is not possible, the relatively long half-life of the tracer allows for regional distribution from one center to surrounding centers, like for [^18^F]FDG.

## Conclusion

We have demonstrated that [^18^F]FDGal PET/CT of the liver can be used to define a radiation dose–response relationship between regional metabolic liver function and radiation dose in patients undergoing SBRT for liver tumors and for evaluation of metabolic recovery. The mean TD_50_ was 17.8 Gy with a linear dose-relationship up to approximately 30 Gy. The development of new methods, such as functional [^18^F]FDGal PET/CT used in the present study, is a promising tool for improving the personalized treatment planning by including variation in regional metabolic liver function (inverse dose-painting), thereby sparing the best functioning sub-volumes of the liver and thereby potentially reduce the risk of RILD.

## Data Availability

The datasets used and/or analyzed during the current study are available from the corresponding author on reasonable request.

## Abbreviations

CI: Confidence interval
CTV: Clinical target volume
CT: Computed tomography
[^18^F]FDGal: 2-Deoxy-2-[^18^F]fluoro-d-galactose
GTV: Gross tumor volume
IHC: Intrahepatic cholangiocarcinoma
*K*_met_ (mL blood/min/mL liver tissue): Hepatic systemic clearance of [^18^F]FDGal
mRC: Metastatic colorectal cancer
PET: Positron emission tomography
PTV: Planning target volume
RFA: Radiofrequency ablation
RILD: Radiation-induced liver disease
SBRT: Stereotactic body radiotherapy
